# Mid-Left Ventricular Ballooning Variant Takotsubo Syndrome Induced by Treadmill Exercise Stress Testing

**DOI:** 10.1155/2018/5282747

**Published:** 2018-11-15

**Authors:** Gregg Cantor, Getu Teressa

**Affiliations:** Department of Medicine, Stony Brook University Medical Center, USA

## Abstract

Stress-induced cardiomyopathy, also known as takotsubo cardiomyopathy, presents similar to a myocardial infarction after a physical or emotional stressor but without any evidence of obstructive coronary artery disease. Different patterns of myocardial involvement and several triggering events have been reported, but classically this condition is characterized by a stress-induced transient left ventricular apical systolic dysfunction. We describe a case of treadmill exercise stress testing-triggered variant of takotsubo cardiomyopathy with mid-left ventricular hypokinesis.

## 1. Introduction

Stress-induced cardiomyopathy, also known as takotsubo cardiomyopathy, is classically characterized by a stress-induced transient left ventricular apical systolic dysfunction, electrocardiographic (EKG) abnormalities, and modestly elevated cardiac enzymes, mimicking myocardial infarction, but in the absence of obstructive coronary artery disease (CAD). Postmenopausal women make up 90% of reported cases, and this condition constitutes up to 0.7-2.5% of cases evaluated for acute coronary syndrome (ACS) [1]. Different patterns of myocardial involvement and several triggering events are being reported. We report the first case of treadmill exercise stress testing-triggered variant of takotsubo cardiomyopathy with mid-left ventricular hypokinesis in a 77-year-old female in which angiography and intravascular ultrasound (IVUS) demonstrated no obstructive CAD or ruptured plaques.

## 2. Case Description

A 77-year-old female with hypertension, untreated hyperlipidemia, hypothyroidism, but without prior history of CAD or angina symptoms was referred to a cardiologist's office for a treadmill exercise test secondary to new onset palpitations. She denied any chest pain or pressure, shortness of breath, exertional dyspnea, or leg swelling. She quit smoking 36 years ago and has no family history of early cardiovascular diseases. She has a very distant cardiac work-up years ago, including a stress test and an echocardiogram, which the patient reported were unremarkable. Vital signs prior to the test were a blood pressure of 140/78, heart rate of 80, and a respiratory rate of 14. Physical exam was unremarkable except for a systolic ejection murmur that was graded II/VI at the base. EKG was at baseline with a normal sinus rhythm, normal axis, and occasional premature ventricular complexes (PVCs).

The patient underwent an exercise stress test using the Bruce protocol and was able to complete stage 1 with exercise for three minutes at a speed of 1.7 mph and a 10% incline. The test was terminated due to dyspnea and fatigue without chest pain. She reached a heart rate of 141 beats per minute which was 98% of predicted for her age. She accomplished 4.5 metabolic equivalents of exertion. With exercise, she had occasional atrial premature complexes and PVCs with a ventricular couplet in recovery. She started to notice tightness in her chest. Her peak blood pressure at the time was 218/90.

**Figure 1:**
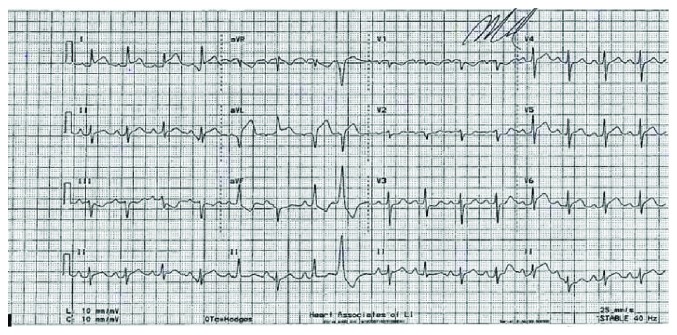
Subsequent EKG.

The patient was transferred onto a stretcher, and an IV line was started. She was given sublingual nitroglycerin, 325 mg of aspirin to chew, and one 5 mg IV push of metoprolol tartrate. She then received nitroglycerin paste and metoprolol tartrate IV every 5 min for two more doses. At that time, her EKG on the stretcher showed ST elevations in leads I, aVL, V5, and V6 with ST depressions in leads III, aVF, and V1-V3 consistent with a lateral wall evolving myocardial infarction (Figure 1). She was transferred urgently to our institution for cardiac catheterization.

**Figure 2:**
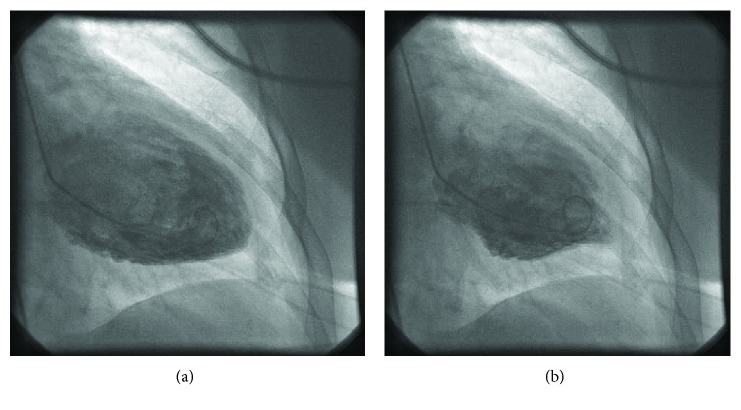
(a) Left ventriculogram: diastole and (b) left ventriculogram: systole.

The patient underwent an emergent cardiac catheterization with left ventriculography and intravascular ultrasound (IVUS) within 2 hours after onset of symptoms. Troponin-I levels prior to the catheterization increased to 11.17 (normal less than 0.05 ng/ml). The rest of the laboratories were within normal limits including a thyroid-stimulating hormone (TSH) level. Coronary angiography showed nonobstructive coronary artery disease (pLAD 40%) and highly tortuous coronary arteries. IVUS of the proximal LAD revealed a minimal lumen area of 5.2mm^2^, and no ruptured plaques. Left ventriculogram revealed a left ventricular ejection fraction (LVEF) of 20% and severe mid-cavitary hypokinesis with basal and apical hyperkinesis (Figures 2(a) and 2(b)). To our knowledge, this is the first case of treadmill exercise testing-triggered mid-left ventricular ballooning variant of takotsubo cardiomyopathy, whereby obstructive epicardial CAD and ruptured plaques were excluded with angiography and IVUS, respectively.

The patient was started on medical management with standard therapy for heart failure. A follow-up echocardiogram was done two days after the event which redemonstrated mid-left ventricular ballooning, with an improved LVEF of 35%. The patient remained asymptomatic during the course of her hospitalization and troponin levels trended down from a postcardiac catheterization peak of 16.06 ng/ml. An echocardiogram was repeated during an outpatient follow-up two weeks later which showed resolution of wall motion abnormalities and an LVEF of 45-50%.

## 3. Discussion

Takotsubo cardiomyopathy is classically characterized by transient left ventricular apical ballooning in the presence of normal or nonobstructive CAD. Patients with transient left ventricular dysfunction usually present with acute onset of chest pain at rest and shortness of breath, along with ST segment elevation, nonspecific ST-T wave changes, and a mild increase in cardiac enzymes, making it indistinguishable from an acute myocardial infarction [1]. The incidence of this pathology is around 2% in patients presenting with acute coronary syndrome and a positive troponin level [2]. The diagnosis of takotsubo cardiomyopathy requires demonstration of normal epicardial coronaries and the absence of ruptured plaques [2–4]. Because of the limited use of cardiac catheterizations and IVUS, the incidence is likely underreported and the condition is therefore misdiagnosed. As the pathophysiology of the stress-induced cardiomyopathy is yet elusive to date, many patients with acute coronary syndrome-like presentations are subjected to treatments with unknown utility for the condition.

Many hypotheses have been proposed to define the pathophysiology, including catecholamine-mediated cardiotoxicity, microvascular dysfunction, coronary vasospasm, oxidative stress, endothelial dysfunction, estrogen deficiency, and cardiac autonomic imbalance [5, 6]. Although catecholamine-mediated cardiotoxicity is one of the most widely proposed mechanisms, given that patients typically present with a preceding history of extreme psychological and/or physical distress, the mechanism of this toxicity is far from being fully elucidated [7]. This mechanism of catecholamine-mediated toxicity has shown to have conflicting results, as some cases report elevated catecholamine levels in takotsubo cardiomyopathy, whereas others show catecholamine levels to have been normal [8]. Due to this, it is unclear if individuals vulnerable to takotsubo cardiomyopathy have heightened catecholamine release in response to stress or have an increased sensitivity to catecholamines than the general population undergoing similar stressful conditions. Another possibility is consistent with the predominance of takotsubo in postmenopausal women which is that a decrease in estrogen decreases the enhanced transcription of cardioprotective factors such as heat shock protein and atrial natriuretic peptide, which, in turn, would make these individuals more susceptible to the toxic effects of catecholamines [7]. However, given the heterogeneous manifestations and triggering events of takotsubo cardiomyopathy, a single mechanistic explanation, such as catecholamine-mediated toxicity, may not adequately explain its pathogenesis.

In conclusion, this case demonstrates treadmill exercise stress testing-triggered variant of takotsubo cardiomyopathy involving the mid-left ventricle in a patient with no demonstrable obstructive CAD nor ruptured plaques. There are multiple cases which report exercise-triggered classic takotsubo cardiomyopathy with apical dyskinesis without a significant decrease in ejection fraction [9–11]. Due to this, our case is unique not just for the involvement of the mid-left ventricle but also that it involved a significant drop in LVEF.
